# Supplementary material for: In Silico Identification and Validation of Organic Triazole Based Ligands as Potential Inhibitory Drug Compounds of SARS-CoV-2 Main Protease
Source: Molecules. 2021 Oct 14;26(20):6199. doi: 10.3390/molecules26206199 (PMC8541586; doi:10.3390/molecules26206199)
Supplement: Supplementary file 1 [file molecules-26-06199-s001.zip › molecules-1396478-supplementary.pdf]

Supplementary Information File

In silico identification and validation of organic triazole based ligands as potential inhibitory drug compounds of SARS-CoV-2 main protease

Vishma Pratap Sur[1], Madhab Kumar Sen[2], Kateřina Komrsková[1, 3*]

[1]Laboratory of Reproductive Biology, Institute of Biotechnology of the Czech Academy of Sciences, BIOCEV, Prumyslova 595, 25250 Vestec, Czech Republic. vishmapratap.sur@ibt.cas.cz

[2]Department of Agroecology and Crop Production, Faculty of Agrobiology, Food and Natural Resources, Czech University of Life Sciences Prague, Kamycka 1176, Prague 6, 165 00, Suchdol, Czech Republic. senm@af.czu.cz

[3]Department of Zoology, Faculty of Science, Charles University, Vinicna 7, 12844 Prague 2, Czech Republic. katerina.komrskova@ibt.cas.cz

* Author to whom correspondence should be addressed.

Table S1 **List of Viruses used for triazole based ligand's antiviral activity screening [1]**

Sr No.	Name of Viruses
1	Human immunodeficiency virus (HIV)
2	Hepatitis C virus (HCV)
3	Hepatitis B virus (HBV)
4	Human herpesvirus
5	Dengue virus
6	Enterovirus Human
7	adenovirus Human
8	cox B1
9	Human echovirus 9
10	Human enterovirus
11	Human polio virus
12	Human rhinovirus
13	Human T lymphotropic virus
14	Influenza A
15	Influenza A (H1N1)
16	Influenza B
17	Monkeypox virus
18	Respiratory syncytial virus
19	Rift Valley fever virus (Cercopithecidae)
20	Sandfly fever Sicilian virus
21	SARS coronavirus
22	Simian virus
23	Sindbis virus
24	Vaccinia virus WR
25	Variola virus
26	Vesicular stomatitis virus
27	West Nile virus
28	Yellow fever virus
29	Human cox B5
30	Human echovirus 13

Table S2 **List of interacting residues participating in M^{pro} ligand pocket formation**

PocID	Chain	SeqID	AA	Atom
1	A	22	CYS	C
1	A	22	CYS	O
1	A	22	CYS	SG
1	A	23	GLY	CA
1	A	24	THR	N
1	A	24	THR	OG1
1	A	25	THR	CB
1	A	25	THR	OG1
1	A	25	THR	CG2
1	A	26	THR	O
1	A	27	LEU	CB
1	A	27	LEU	CD2
1	A	41	HIS	O
1	A	41	HIS	ND1
1	A	41	HIS	CD2
1	A	41	HIS	CE1
1	A	41	HIS	NE2
1	A	42	VAL	O
1	A	43	ILE	CA
1	A	43	ILE	C
1	A	43	ILE	O
1	A	44	CYS	C
1	A	44	CYS	O
1	A	44	CYS	CB
1	A	45	THR	CA
1	A	45	THR	C
1	A	45	THR	O
1	A	45	THR	CG2
1	A	46	SER	N
1	A	46	SER	CA
1	A	46	SER	O
1	A	46	SER	CB
1	A	46	SER	OG
1	A	49	MET	CB

1	A	49	MET	CG
1	A	49	MET	CE
1	A	61	LYS	CE
1	A	61	LYS	NZ
1	A	140	PHE	C
1	A	140	PHE	O
1	A	140	PHE	CB
1	A	141	LEU	CA
1	A	141	LEU	C
1	A	141	LEU	O
1	A	142	ASN	N
1	A	142	ASN	CA
1	A	142	ASN	OD1
1	A	143	GLY	N
1	A	143	GLY	CA
1	A	144	SER	N
1	A	144	SER	OG
1	A	145	CYS	CB
1	A	145	CYS	SG
1	A	163	HIS	NE2
1	A	164	HIS	O
1	A	164	HIS	CB
1	A	165	MET	CA
1	A	165	MET	CB
1	A	165	MET	SD
1	A	165	MET	CE
1	A	166	GLU	N
1	A	166	GLU	O
1	A	166	GLU	CB
1	A	166	GLU	OE2
1	A	167	LEU	CA
1	A	167	LEU	CD2
1	A	168	PRO	CG
1	A	168	PRO	CD
1	A	181	PHE	CZ
1	A	186	VAL	O

1	A	187	ASP	CA
1	A	187	ASP	C
1	A	187	ASP	CB
1	A	188	ARG	N
1	A	188	ARG	CA
1	A	188	ARG	C
1	A	188	ARG	O
1	A	189	GLN	N
1	A	189	GLN	CA
1	A	189	GLN	CB
1	A	189	GLN	OE1
1	A	189	GLN	NE2
1	A	190	THR	N
1	A	190	THR	O
1	A	192	GLN	O
1	A	192	GLN	CG
1	A	192	GLN	NE2

Table S3 **List of best ligand molecules according to their binding affinity score through docking process**

DrugBank ligand Chemical ID	Binding affinity values (kcal/mole)
**DB12411**	**-10.2**
**DB11262**	**-9**
**DB07213**	**-8.8**
**DB07020**	**-8.8**
DB04285	-8.7
DB15396	-8.7
DB06295	-8.7
DB03231	-8.6
DB11652	-8.6
DB15310	-8.5
DB13993	-8.5
DB12886	-8.4
DB12895	-8.4
DB12740	-8.4
DB08006	-8.4
DB02651s	-8.4
DB03005	-8.4
DB00673	-8.2
DB03571	-8.2
DB07359	-8.2
DB11760	-8.2
DB11942	-8.2
DB12694	-8.2
DB15190	-8.2
DB13113	-8.1
DB12848	-8
DB07072	-8

Table S4 **Evaluation of Lipinski's rule of 5 with a drug-likeness score by Molsoft LLC: Drug-Likeness and molecular property prediction of the selected molecules (best 4 ligands)**

**Compounds**	**Mass (<500)**	**Hydrogen bond acceptor (<10)**	**Hydrogen bond donor (<5)**	**LOGP (<5)**	**Molar Refractivity (40-130)**	**Drug likeness (>0)**
Bemcentinib (DB12411)	506.29	5	3	5.42	80.73	0.06
Bisoctrizole (DB11262)	658.40	6	2	13.41	78.39	-0.59
PYIITM (DB07213)	427.21	5	4	4.08	91.08	0.38
NIPFC (DB07020)	370.12	5	3	3.54	90.31	0.07

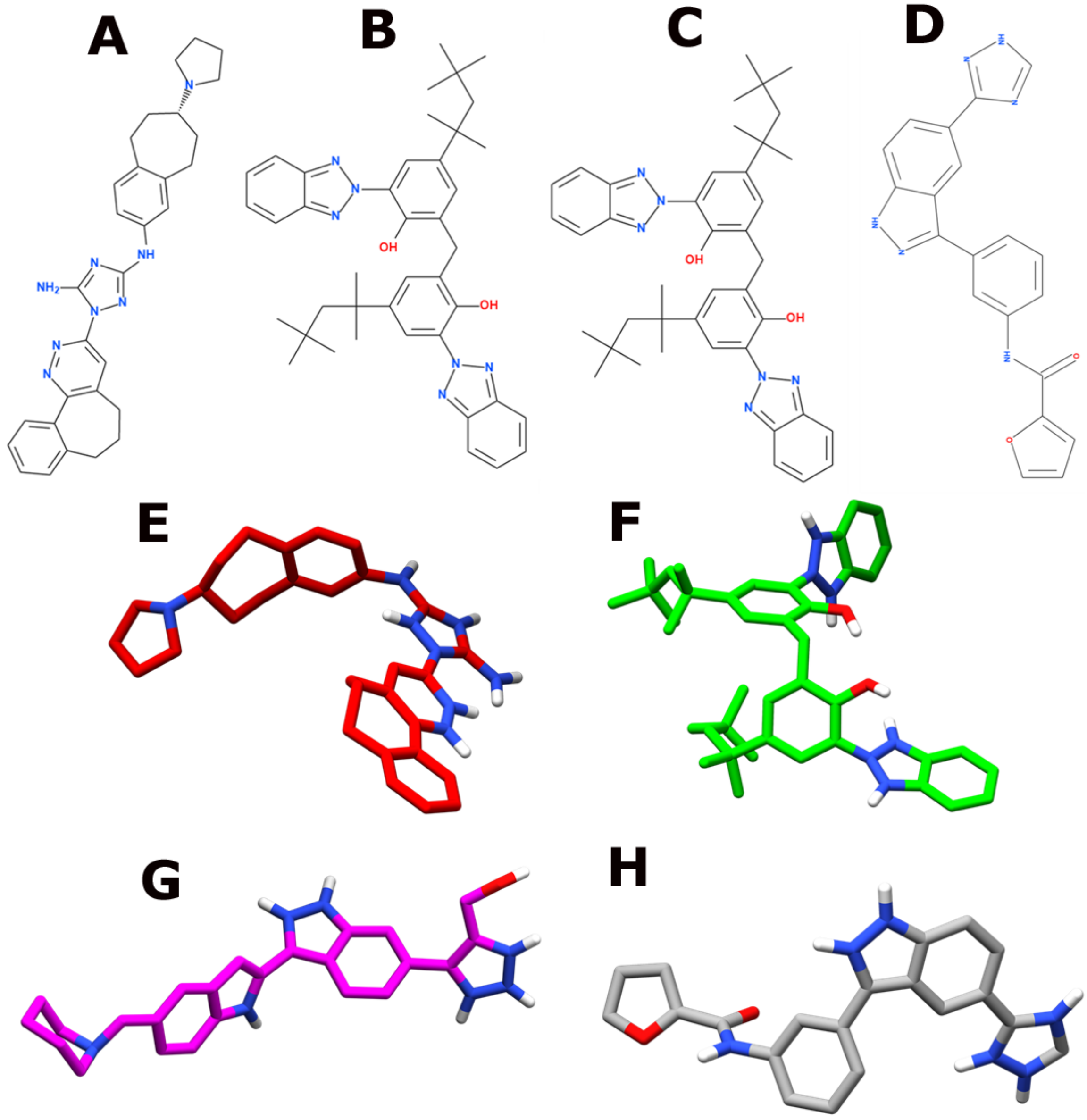

Figure S1 2D and 3D chemical structure of the best 4 triazole based organic ligands

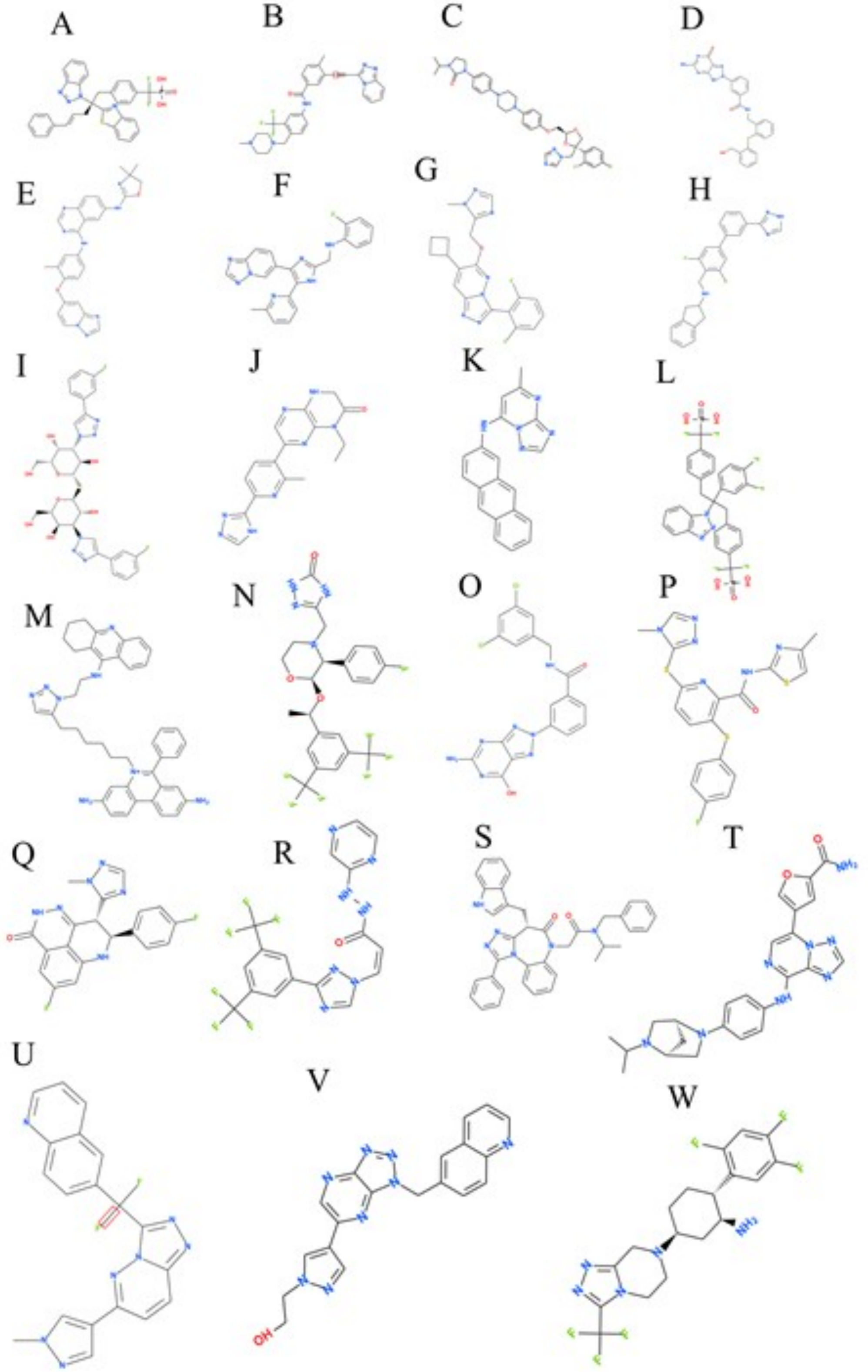

Figure S2 **2D chemical structure of the best 23 triazole based organic ligands**

Table S5: Ligands already used as M^{pro} inhibitor, used as a reference with triazole ligands docking study.

Ligand	Binding Affinity (kcal/mol)
11a	-7.2
11b	-7.5

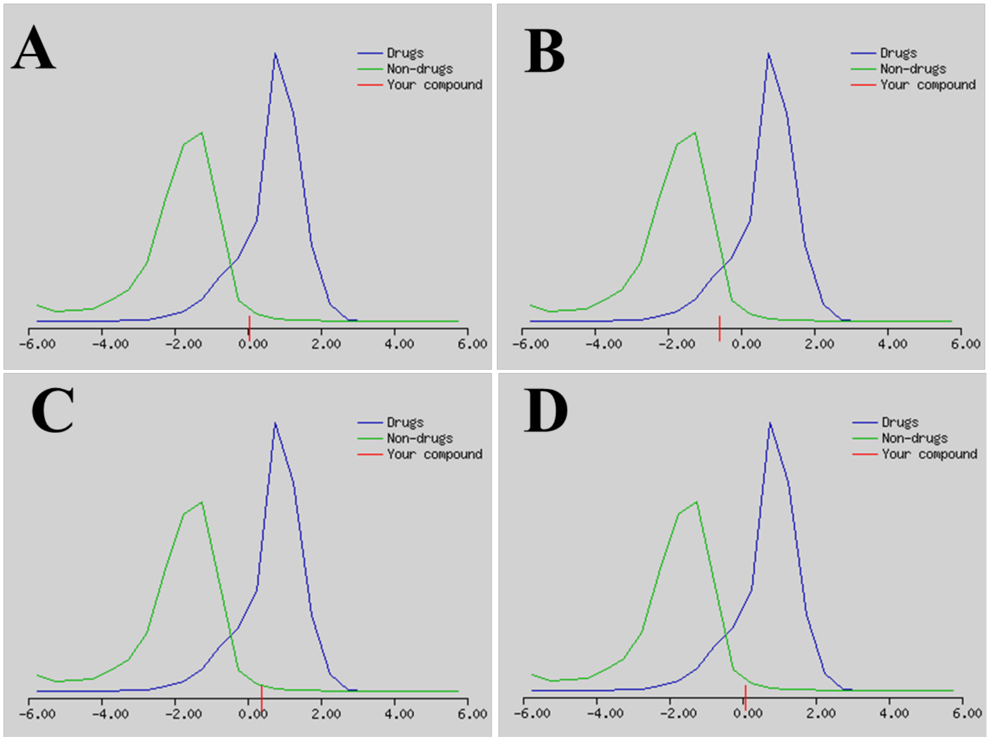

Figure S3 **Drug likeness evaluation of selected ligands using Molsoft LLC: Drug-Likeness and molecular property prediction. Bemcentinib (DB12411) (A), Bisoctrizole (DB11262) (B), PYIITM (DB07213) (C), and NIPFC (DB07020) (D)**

Table S6 **Triazole based organic ligands antiviral activity screening through web based antiviral compound prediction server**

Query molecule	General (%)	HBV (%)	HCV (%)	HHV (%)	HIV (%)
Bemcentinib	50.34	21.78	61.46	37.85	60.71
Bisoctriazole	61.38	21.54	70.42	30.32	60.32
PYIITM	62.49	23.78	32.55	95.14	48.11
NIPFC	36.00	28.59	36.52	39.13	60.61

Script S1 NVT run:

Title = Mpro-ligand complex NVT equilibration

Define = -DPOSRES ; position restrain the protein and ligand

Run parameters

Integrator = md ; leap-frog integrator

Nsteps = 50000 ; 2 * 50000 = 100 ps

Dt = 0.002 ; 2 fs

Output control

Nstenergy = 500 ; save energies every 1.0 ps Nstlog

= 500 ; update log file every 1.0 ps Nstxout-

compressed = 500 ; save coordinates every 1.0 ps

Bond parameters

Continuation = no ; first dynamics run

Constraint_algorithm = lincs ; holonomic constraints

Constraints = h-bonds ; bonds to H are constrained

Lincs_iter = 1 ; accuracy of LINCS

Lincs_order = 4 ; also related to accuracy

Neighbor searching and vdW

Cutoff-scheme = Verlet

Ns_type = grid ; search neighboring grid cells

Nstlist = 20 ; largely irrelevant with Verlet

Rlist = 1.2

Vdwtype = cutoff

Vdw-modifier = force-switch

Rvdw-switch = 1.0

Rvdw = 1.2 ; short-range van der Waals cutoff (in nm)

Electrostatics

Coulombtype = PME ; Particle Mesh Ewald for long-range electrostatics

Rcoulomb = 1.2 ; short-range electrostatic cutoff (in nm)

Pme_order = 4 ; cubic interpolation

Fourierspacing = 0.16 ; grid spacing for FFT

Temperature coupling

Tcoupl = V-rescale ; modified Berendsen thermostat

Tc-grps = Protein_lig1 Water and_ions ; two coupling groups - more accurate

Tau_t = 0.1 0.1 ; time constant, in ps

Ref_t = 300 300 ; reference temperature, one for each group, in K

Pressure coupling

Pcoupl = no ; no pressure coupling in NVT

Periodic boundary conditions

Pbc = xyz ; 3-D PBC

Dispersion correction is not used for proteins with the C36 additive FF

DispCorr = no

Velocity generation

Gen_vel = yes ; assign velocities from Maxwell distribution

Gen_temp = 300 ; temperature for Maxwell distribution

Gen_seed = -1 ; generate a random seed

Script S2 NPT run:

Title = Mpro-ligand complex NPT equilibration

Define = -DPOSRES ; position restrain the protein and ligand

Run parameters

Integrator = md ; leap-frog integrator

Nsteps = 50000 ; 2 * 50000 = 100 ps

Dt = 0.002 ; 2 fs

Output control

Nstenergy = 500 ; save energies every 1.0 ps

Nstlog = 500 ; update log file every 1.0 ps

Nstxout-compressed = 500 ; save coordinates every 1.0 ps

Bond parameters

Continuation = yes ; continuing from NVT

Constraint_algorithm = lincs ; holonomic constraints

Constraints = h-bonds ; bonds to H are constrained

Lincs_iter = 1 ; accuracy of LINCS

Lincs_order = 4 ; also related to accuracy

Neighbor searching and vdW

Cutoff-scheme = Verlet

Ns_type = grid ; search neighboring grid cells

Nstlist = 20 ; largely irrelevant with Verlet

Rlist = 1.2

Vdwtype = cutoff

Vdw-modifier = force-switch

Rvdw-switch = 1.0

Rvdw = 1.2 ; short-range van der Waals cutoff (in nm)

Electrostatics

Coulombtype = PME ; Particle Mesh Ewald for long-range electrostatics

Rcoulomb = 1.2

Pme_order = 4 ; cubic interpolation

Fourierspacing = 0.16 ; grid spacing for FFT

Temperature coupling

Tcoupl = V-rescale ; modified Berendsen thermostat

Tc-grps = Protein_lig1 Water_and_ions ; two coupling groups - more accurate

Tau_t = 0.1 0.1 ; time constant, in ps

Ref_t = 300 300 ; reference temperature, one for each group, in K

Pressure coupling

Pcoupl = Berendsen ; pressure coupling is on for NPT

Pcoupltype = isotropic ; uniform scaling of box vectors

Tau_p = 2.0 ; time constant, in ps

Ref_p = 1.0 ; reference pressure, in bar

Compressibility = 4.5e-5 ; isothermal compressibility of water, bar^-1

Refcoord_scaling = com

Periodic boundary conditions

Pbc = xyz ; 3-D PBC

Dispersion correction is not used for proteins with the C36 additive FF

DispCorr = no

Velocity generation

gen_vel = no ; velocity generation off after NVT

Script S3 MD run :

title = OPLS Mpro NPT equilibration

Run parameters

integrator = md ; leap-frog integrator

nsteps = 20000000 ; 2 * 20000000 = 40000 ps (40 ns)

Dt = 0.002 ; 2 fs

Output control

nstxout = 0 ; suppress bulky .trr file by specifying

nstvout = 0 ; 0 for output frequency of nstxout,

nstfout = 0 ; nstvout, and nstfout

nstenergy = 5000 ; save energies every 10.0 ps

nstlog = 5000 ; update log file every 10.0 ps

nstxout-compressed = 5000 ; save compressed coordinates every 10.0 ps

compressed-x-grps = System ; save the whole system

Bond parameters

Continuation = yes ; Restarting after NPT

Constraint_algorithm = lincs ; holonomic constraints

Constraints = h-bonds ; bonds involving H are constrained

Lincs_iter = 1 ; accuracy of LINCS

Lincs_order = 4 ; also related to accuracy

Neighbor searching

Cutoff-scheme = Verlet ; Buffered neighbor searching

Ns_type = grid ; search neighboring grid cells

Nstlist = 10 ; 20 fs, largely irrelevant with Verlet scheme

Rcoulomb = 1.0 ; short-range electrostatic cutoff (in nm)

Rvdw = 1.0 ; short-range van der Waals cutoff (in nm)

Electrostatics

Coulombtype = PME ; Particle Mesh Ewald for long-range electrostatics

Pme_order = 4 ; cubic interpolation

Fourierspacing = 0.16 ; grid spacing for FFT

Temperature coupling is on

Tcoupl = V-rescale ; modified Berendsen thermostat

Tc-grps = Protein Non-Protein ; two coupling groups - more accurate

Tau_t = 0.1 0.1 ; time constant, in ps

Ref_t = 300 300 ; reference temperature, one for each group, in K

Pressure coupling is on

Pcoupl = Parrinello-Rahman ; Pressure coupling on in NPT

Pcoupltype = isotropic ; uniform scaling of box vectors

Tau_p = 2.0 ; time constant, in ps

Ref_p = 1.0 ; reference pressure, in bar

Compressibility = 4.5e-5 ; isothermal compressibility of water, bar^-1

Periodic boundary conditions Pbc

= xyz ; 3-D PBC

Dispersion correction

DispCorr = EnerPres ; account for cut-off vdW scheme

Velocity generation

Gen_vel = no ; Velocity generation is off

Script 4 Interaction energy run

Title = Mpro-ligand complex MD simulation

Run parameters

Integrator = md ; leap-frog integrator

Nsteps = 5000000 ; 2 * 5000000 = 10000 ps (10 ns)

Dt = 0.002 ; 2 fs

Output control

Nstenergy = 5000 ; save energies every 10.0 ps Nstlog

= 5000 ; update log file every 10.0 ps

Nstxout-compressed= 5000 ; save coordinates every 10.0 ps

Energygrps = Protein lig1

Bond parameters

Continuation = yes ; continuing from NPT

Constraint_algorithm = lincs ; holonomic constraints

Constraints = h-bonds ; bonds to H are constrained

Lincs_iter = 1 ; accuracy of LINCS

Lincs_order = 4 ; also related to accuracy

Neighbor searching and vdW

Cutoff-scheme = Verlet

Ns_type = grid ; search neighboring grid cells

Nstlist = 20 ; largely irrelevant with Verlet

Rlist = 1.2

Vdwtype = cutoff

Vdw-modifier = force-switch

Rvdw-switch = 1.0

Rvdw = 1.2 ; short-range van der Waals cutoff (in nm)

Electrostatics

Coulombtype = PME ; Particle Mesh Ewald for long-range electrostatics

Rcoulomb = 1.2

Pme_order = 4 ; cubic interpolation

Fourierspacing = 0.16 ; grid spacing for FFT

Temperature coupling

Tcoupl = V-rescale ; modified Berendsen thermostat

Tc-grps = Protein_lig1 Water_and_ions ; two coupling groups - more accurate

Tau_t = 0.1 0.1 ; time constant, in ps

Ref_t = 300 300 ; reference temperature, one for each group, in K

Pressure coupling

Pcoupl = Parrinello-Rahman ; pressure coupling is on for NPT

Pcoupltype = isotropic ; uniform scaling of box vectors

Tau_p = 2.0 ; time constant, in ps

Ref_p = 1.0 ; reference pressure, in bar

Compressibility = 4.5e-5 ; isothermal compressibility of water, bar^-1

Periodic boundary conditions

Pbc = xyz ; 3-D PBC

Dispersion correction is not used for proteins with the C36 additive FF

DispCorr = no

Velocity generation

Gen_vel = no ; continuing from NPT equilibration

Reference

1. Qureshi, A.; Kaur, G.; Kumar, M. AVCpred: an integrated web server for prediction and design of antiviral compounds. *Chem Biol Drug Des* **2017**, *89*, 74-83, doi:10.1111/cbdd.12834.
